# Offending behaviour, health and wellbeing of military veterans in the criminal justice system

**DOI:** 10.1371/journal.pone.0207282

**Published:** 2018-11-09

**Authors:** Roxanna Short, Hannah Dickson, Neil Greenberg, Deirdre MacManus

**Affiliations:** 1 Forensic and Neurodevelopmental Sciences, Institute of Psychiatry, Psychology and Neuroscience, King’s College London, London, United Kingdom; 2 King’s Centre for Military Health Research, King’s College London, London, United Kingdom; Uniformed Services University of the Health Sciences, UNITED STATES

## Abstract

**Background:**

A small but significant proportion of military veterans become involved in the criminal justice system (CJS) after leaving service. Liaison and Diversion (L&D) services aim to identify vulnerable offenders in order to provide them with the health/welfare support they need, and (where possible) divert them away from custody. An administrative database of L&D service-users was utilised to compare the needs of veterans with those of non-veteran L&D service-users.

**Method:**

National data collected from 29 L&D services in 2015–2016 was utilised. Of the 62,397 cases, 1,067 (2%) reported previous service in the Armed Forces, and 48,578 had no previous service history. The associations between veteran status and socio-demographic characteristics, offending behaviour, health- and mental health-problems were explored. The associations between specific types of offending and mental health problems within the veterans in the sample were also investigated.

**Results:**

Veterans tended to be older, and less likely to be unemployed than non-veterans, but just as likely to have unstable living arrangements (including homelessness). Veteran status was associated with increased levels of interpersonal violence, motoring offences, anxiety disorders and hazardous drinking patterns. Veteran status was associated with decreased levels of acquisitive offending, schizophrenia, ADHD, and substance misuse. Among veterans, the presence of an anxiety disorder (umbrella term which included GAD, Phobias, PTSD etc.) was associated with increased interpersonal violence, alcohol misuse was associated with increased motoring offences, and substance use was associated with increased acquisitive offending.

**Conclusions:**

Our study indicates that among offenders in the CJS who have been identified as needing health or welfare support, veterans differ from non-veterans in terms of their health and welfare needs and offending behaviour. These differences may be influenced by the impact of military service and the transition into civilian life. Our findings support the identification of military personnel within the CJS to provide appropriate interventions and support to improve outcomes and reduce offending.

## Introduction

The majority of service leavers make successful transitions back into civilian life [1]. However, recent research has shown that a minority, who become involved in the Criminal Justice System (CJS), often experience health, behavioural and social problems [2]. Estimates of the proportion of the prison population who have previously served in the UK Armed Forces have ranged from 3.5% to 17% depending on the methodology and sample used [3–5]. Furthermore, it was estimated that 3.4% of adults subject to probation supervision in England and Wales in 2009 were veterans [6]. A government review of ex-Armed Forces Personnel in the CJS [7] highlighted the need for better identification of the needs of veterans in the CJS to inform the development of services to help reduce re-offending. Previous research into the needs of veterans in the CJS has been limited by the use of selected samples from specific areas of the CJS, such as prison or probation, with limited data on health and welfare needs [8].

Government statistics show that veterans form the largest single occupational group within the prison and probation services, and that they are more likely to have committed a violent or sexual offence than offenders who have not served in the military [3,6]. However, these statistics relate to those who are given a custodial sentence or probation supervision order. Many offenders will not receive either category of sentence. Risk factors for offending in veterans are largely similar to those for civilians (i.e. younger age, male gender, lower socioeconomic class, history of offending) [9,10], but with some notable exceptions. Veterans who offend tend to be older, on average, than general population offenders [3]. This may be because military service acts to reduce the opportunity for offending (or the risk of conviction) at a time when young men in the general population are most at risk of offending, and thus the individual’s time in service acts as a “hiatus” from offending that may have occurred anyway [8]. Prior research also indicates that experiences resulting from military service can increase the risk of offending in some individuals [11], suggesting a distinct pathway to offending in this particular group. For example, certain aspects of deployment, such as deploying in a combat role and exposure to trauma during deployment, have been shown to increase the risk of violent offending by military personnel on return [11–13]. Finally, some but not all mental health problems, such as Posttraumatic Stress Disorder (PTSD) and alcohol misuse, have been shown to increase the risk of offending behaviour among military personnel [11,14].

Existing research also suggests that socioeconomic needs, such as relationship problems, financial instability, unemployment and lack of stable accommodation, are strong risk factors for offending among veterans and that stability in these areas can be protective against the risk of offending in the presence of mental health problems [13,15,16]. Therefore, by the identification of socioeconomic and mental health needs among veterans on entry into the CJS we have the potential to inform the development of early intervention services for veterans.

The purpose of Liaison and Diversion (L&D) services is to provide an assessment of individuals within the CJS who have been identified as having a psychosocial need (for example, mental health problems, learning difficulties, physical health problems, alcohol- and substance-use problems, welfare needs), to ensure that they receive the appropriate support, and to (where possible/appropriate) divert them out of the CJS and into health, social care or other services. Referral to L&D services usually occurs at the earliest opportunity, but may take place at any stage of the CJS. Furthermore, referrals can be made by a wide range of agencies, including: police, Crown Prosecution Service, youth offending teams, social workers, drugs/alcohol services, defence lawyers, and parents/guardians/family members. This broad referral process maximises the reach of the L&D service to the most vulnerable of individuals at the earliest opportunity. Individuals referred to L&D services are offered a screening appointment with a mental health practitioner, during which they are asked whether they have ever served in the UK Armed Forces. To date, there has been no formal comparison of L&D service offenders with offenders who have not been referred to these services. However, it is likely that those offenders referred to L&D services represent a particularly vulnerable group of offenders with a range of psychosocial vulnerabilities.

The research to date has focused either on aspects of military service that are risk factors for offending, or comparisons of offending between veterans and non-veterans within the CJS without considering other factors such as health, mental health and welfare needs. Our study aims to address this gap in the literature. We utilised the national L&D database of all offenders, both Armed Forces veterans and non-veterans, who were assessed by the 29 services across England during 2015–2016. Our primary aim was to identify differences between veterans and non-veterans within L&D services, in terms of: socioeconomic and welfare needs; offending behaviour; and health and mental health problems. In addition, we examined factors associated with specific types of offending (interpersonal violence, acquisitive and motoring offences) among veterans referred to L&D services.

## Methods

### Sample and procedure

**Fig 1:**
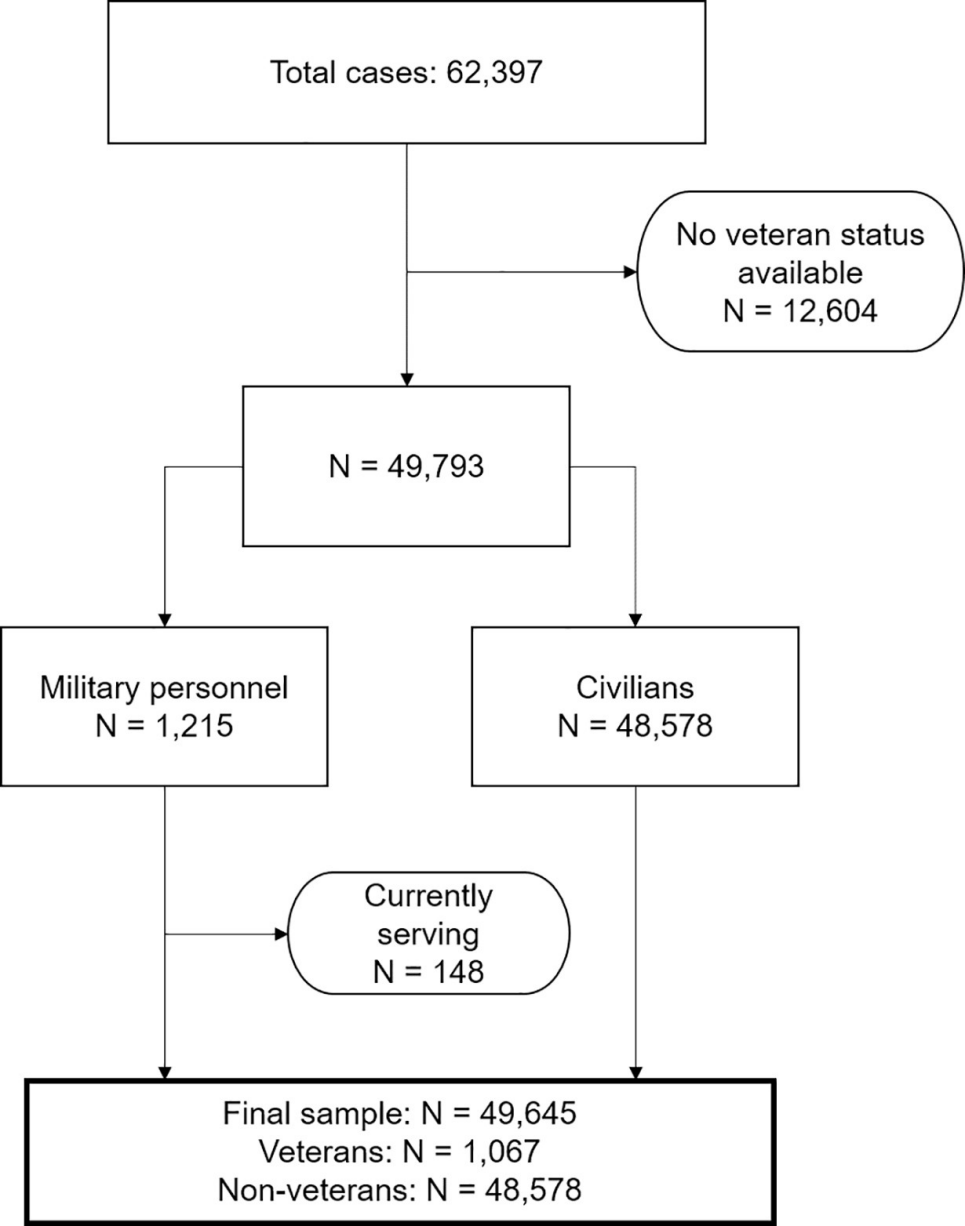
Flow chart illustrating veteran status case selection.

This study employed routinely-collected data from 29 separate L&D services from April 2015 until April 2016. Data were gathered by L&D service practitioners, and included information pertaining to the individual’s: military status; socio-demographics; current offence; mental health needs; alcohol/substance use; and other vulnerabilities (learning, physical, or social and communication difficulties). Data were entered onto the database on a case-by-case basis: each referral was treated as a separate case. Thus, in the absence of an individual identifier, the same individual may have multiple entries relating to different offences. As shown in Fig 1, a total of 62,397 referrals were made to the 29 L&D sites between April 2015 and April 2016. Of these, 49,793 (80%) cases included information regarding the individual’s military status, 1,215 (2.4%) of which pertained to individuals who reported that they had served, or were currently serving, in the UK Armed Forces. Given that the majority of the military personnel in the database were veterans, we categorised the cases with a recorded military status as veterans (N = 1,067; includes individuals who left military service within the last 12 months, 1–5 years ago, or more than 5 years ago), or non-veterans (N = 48,578), and excluded those who reported that they were currently serving (N = 148; see Fig 1).

Permission to access the L&D database was sought from and approved by NHS Digital following ethical approval (reference: LRS15/162992) to ensure all information governance policies and procedures were met. The dataset was fully anonymised before it was accessed.

### Measures

#### Veteran status

In accordance with the UK Government’s criteria, a military veteran was classified by L&D services as anyone who had served for at least one day in the UK Armed Forces, and had left service at the time of contact with L&D services. All other non-military cases were classified as non-veterans. We note that our data are cross-sectional, thus our analyses reflect associations with veteran status, rather than its role as a risk factor for offending or physical/mental health problems.

#### Socio-demographics

Socio-demographic factors included: age, gender, ethnicity (white vs black and minority ethnic; BME), employment status (employed; unemployed; sickness/disability; retired; other), and accommodation status (homeless; in temporary accommodation; in owned/rented accommodation; living with parents/relatives; other). Age was recorded in 5-year increments, and reflected the age-group of the individual at their first meeting with the L&D service. For the purposes of the statistical analyses, age was recoded in to six categories: 25 years and under, 26–35 years, 36 to 45 years, 46 to 55 years, 56 to 65 years, and over 65 years.

#### Current offence

This was recorded as the most serious offence that the individual was charged with, or suspected of having committed, at the time of their referral to the L&D service. Offences were classified as: violence against the person (including murder, manslaughter, violence against the person, harassment, robbery); sex offence; non-interpersonal violence (including criminal damage, arson, possession of an offensive weapon, possession of a firearm); acquisitive offence (including theft, burglary, fraud/forgery); drug offence; public order offence; motoring offence; breach of court order; and other. For the purposes of statistical analyses, this was recoded into nine separate binary variables, each indicating the presence or absence of each offence type, in line with previous research [17]. However, for each of these variables, we are comparing the presence of one offence type with all other offence types (rather than with non-offenders, as we did not have a non-offender comparison group).

#### Alcohol and substance misuse

Both alcohol and substance misuse were assessed using standardised instruments: the Alcohol Use Disorders Identification Test [18] for alcohol misuse; and the Drug Use Disorders Identification Test [19] for substance misuse. Alcohol use was categorised as hazardous (the consumption of over 14 units per week for women, and over 21 units per week for men), harmful (the individual experiences health problems that are directly related to alcohol consumption), or dependent (the individual feels unable to function without using alcohol) [18]. Presence of substance misuse was reflected by evidence of social, occupational, psychological, or physical problems related to use of drugs [19].

#### Wider health needs

The presence of learning difficulties (where suspected) was established using standard cut-off scores on the Learning Disability Screening Questionnaire [20] or the Hayes Ability Screening Index [21]. Presence of social & communication difficulties (where suspected) was established using the standard cut-off score on the Autism Spectrum Quotient [22]. The presence of physical health problems was established via self-report.

#### Mental health

Individuals were screened for, or diagnosed with, the following: schizophrenia, bipolar affective disorder, depression, anxiety disorders (including generalised anxiety, PTSD, phobias, panic disorder, and obsessive-compulsive disorder), adjustment disorder, eating disorders, dementia, attention deficit/hyperactivity disorder (ADHD), and personality disorder. Up to three mental health problems could be recorded for each case. Whilst all mental disorders were assessed using standardised screening tools and information from medical records where available, the screening and diagnostic methods varied among the different sites. As a consequence, some recorded mental disorders reflect actual diagnoses, whereas others reflect elevated scores on screening questionnaires.

### Statistical analyses

We conducted the analyses using a series of univariate and multivariate logistic regression models in Stata 14 [23]. Standard errors were adjusted to account for the arbitrary correlation of errors across cases within each L&D site using Stata’s “cluster” algorithm [24,25]. First, we identified any socio-demographic variables that were independently associated with veteran status using a series of univariate and adjusted logistic regression analyses. These were used as covariates in the subsequent analyses comparing veterans with non-veterans. Second, we examined the univariate and adjusted associations between veteran status and each of the offence type, health needs and mental health variables in separate logistic regression models. Results from the univariate analyses are reported as odds ratios (OR) with 95% confidence intervals (CI), and results from the multivariate analyses are reported as adjusted odds ratios (aOR) with 95% CIs.

### Factors associated with different types of offending among veterans

We then examined the factors associated with particular offence types in the veteran sample (N = 1,067). In these analyses, we only examined offences that were independently associated with veteran status in the preceding analyses: violence against the person (vs other non-violent and non-sexual offences); acquisitive offences (vs all other offence types); and motoring offences (vs all other offence types). As in the preceding analyses, we first identified any socio-demographic variables that were independently associated with each offence type using separate logistic regression analyses. We then conducted a series of logistic regressions to examine the univariate and adjusted associations between each offence type and each of the mental health variables. In these analyses we did not adjust the standard errors based on L&D site due to the small number of veterans per site [26]. First, we established which socio-demographic variables were independently associated with the offence type. These were retained as covariates in the following analyses. Second, we examined the univariate and adjusted associations between each of the mental health and alcohol/substance use variables and the offence type outcome.

As we were unable to identify which cases belonged to separate individuals in the database, and thus unable to assess the extent of clustering in data, we performed a crude sensitivity analysis. We matched the cases on a series of “static” variables (L&D site, gender, age, ethnicity, veteran status, presence of physical disabilities, presence of learning difficulties, and presence of social and communication difficulties), removed the duplicates (by selecting the first case in a cluster), and repeated our analyses. Our main results remained unchanged, therefore we report the results of the analysis of the full dataset.

## Results

### Socio-demographic characteristics

The veterans in the sample were predominantly male, aged 26–35, and of white ethnicity. These characteristics were significantly associated with veteran status (see Table 1). Veterans were more likely than non-veterans to be employed (OR = 2.63, 95% CI 2.25–3.07) or retired (OR = 8.77, 95% CI 6.54–11.75) than unemployed. Veterans were less likely than non-veterans to be living with relatives (vs. being in owned or rented accommodation; OR = 0.69, 95% CI 0.52–0.91). Veterans and non-veterans were equally likely to report being homeless (OR = 0.91, 95% CI 0.69–1.20). In the multivariate logistic regression model, gender, age, ethnicity, and employment status remained independently associated with veteran status (see Table 1), and were retained as covariates in the following multivariate analyses.

**Table 1:** Association of socio-demographic factors with veteran status.

		Non-veterans (N = 48,578)	Veterans (N = 1,067)							
		N (%)	N (%)	OR [95% CI]	p		aOR [95% CI]^†^		p	
Gender										
	Female	11096 (22.84)	33 (3.09)	1	-		1		-	
	Male	37112 (76.40)	1028 (96.34)	9.31 [6.69–12.97]	<0.01		10.44 [7.84–13.91]		<0.01	
Age										
	25 & Under	13385 (27.55)	100 (9.37)	1	-		1		-	
	26–35	15813 (32.55)	328 (30.74)	2.78 [2.12–3.64]	<0.01		2.80 [2.06–3.81]		0.01	
	36–45	10575 (21.77)	277 (25.96)	3.51 [2.65–4.63]	<0.01		3.68 [2.64–5.12]		0.01	
	46–55	6276 (12.92)	214 (20.06)	4.56 [3.20–6.51]	<0.01		4.68 [3.20–6.82]		0.01	
	56–65	1896 (3.90)	72 (6.75)	5.08 [3.39–7.61]	<0.01		3.92 [2.40–6.39]		0.01	
	Over 65	423 (0.87)	71 (6.65)	22.47 [14.78–34.14]	<0.01		9.70 [6.09–15.46]		0.01	
Ethnicity										
	BME	6625 (13.64)	65 (6.09)	1	-		1		-	
	White	40981 (84.36)	974 (91.28)	2.42 [1.73–3.40]	<0.01		2.48 [1.88–3.27]		0.01	
Employment status										
	Employed	8239 (16.96)	337 (31.58)	2.63 [2.25–3.07]	<0.01		2.42 [2.03–2.89]		0.01	
	Unemployed	29104 (59.91)	453 (42.46)	1	-		1		-	
	Sickness/disability	5497 (11.32)	117 (10.97)	1.37 [0.95–1.98]	0.10		1.20 [0.84–1.73]		0.32	
	Retired	601 (1.24)	82 (7.69)	8.77 [6.54–11.75]	<0.01		3.62 [2.41–5.43]		0.01	
	Other	1659 (3.42)	24 (2.25)	0.93 [0.58–1.48]	0.76		1.51 [0.92–2.50]		0.10	
Accommodation status										
	Homeless	4481 (9.22)	98 (9.18)	0.91 [0.69–1.20]	0.51		1.07 [0.83–1.39]		0.59	
	Temporary	4234 (8.72)	73 (6.84)	0.72 [0.49–1.05]	0.08		0.95 [0.66–1.38]		0.79	
	Own/Rent	26603 (54.76)	638 (59.79)	1	-		1		-	
	Parent/Family	7445 (15.33)	123 (11.53)	0.69 [0.52–0.91]	<0.01		0.93 [0.68–1.27]		0.63	
	Other	1782 (3.67)	38 (3.56)	0.89 [0.58–1.37]	0.60		0.96 [0.66–1.40]		0.83	

^†^ Adjusted odds ratios: adjusted for age, gender, ethnicity, employment status and accommodation status.

### Offence type

Offences classed as violence against the person were the most common type of offending among the whole sample (32%), followed by acquisitive (16%), public order (11%), and non-interpersonal violence offences (10%). The remaining offence types were relatively uncommon. As shown in Table 2, veterans were more likely to have committed violence against the person (OR = 1.28, 95% CI 1.10–1.49) or motoring offences (OR = 1.85, 95% CI 1.40–2.43), and less likely to have committed acquisitive offences (OR = 0.56, 95% CI 0.43–0.74) than non-veterans. These differences remained significant after adjusting for age, gender, ethnicity and employment status (see Table 2). Veterans were also more likely to have committed a sexual offence than non-veterans (OR = 1.54, 95% CI 1.10–2.14), but the multivariate model suggests that this association was driven by differences in socio-demographic characteristics (aOR = 0.77, 95% CI 0.54–1.10). The remaining offence types (drug, public order and breach offences) were not independently associated with veteran status.

**Table 2:** Association of offence type with veteran status.

		Non-veterans (N = 48,578)	Veterans (N = 1,067)				
		N (%)	N (%)	OR [95% CI]	p	aOR [95% CI]^†^	p
Violence against the person							
	No	32227 (66.34)	645 (60.45)	1	-	1	-
	Yes	15410 (31.72)	396 (37.11)	1.28 [1.10–1.49]	<0.01	1.36 [0.94–1.99]	0.11
Sex							
	No	45125 (92.89)	959 (89.88)	1	-	1	-
	Yes	2512 (5.17)	82 (7.69)	1.54 [1.10–2.14]	0.01	1.11 [0.58–2.15]	0.75
Acquisitive							
	No	39844 (82.02)	938 (87.91)	1	-	1	-
	Yes	7793 (16.04)	103 (9.65)	0.56 [0.43–0.74]	<0.01	1.42 [1.05–1.92]	0.02
Violence							
	No	42662 (87.82)	953 (89.32)	1	-	1	-
	Yes	4975 (10.24)	88 (8.25)	0.79 [0.62–1.01]	0.06	1.03 [0.61–1.75]	0.91
Motoring							
	No	45501 (93.67)	958 (89.78)	1	-	1	-
	Yes	2136 (4.40)	83 (7.78)	1.85 [1.40–2.43]	<0.01	0.39 [0.16–0.94]	0.03
Drugs							
	No	45834 (94.35)	1010 (94.66)	1	-	1	-
	Yes	1803 (3.71)	31 (2.91)	0.78 [0.55–1.11]	0.16	1.31 [0.96–1.78]	0.08
Public order							
	No	42495 (87.48)	933 (87.44)	1	-	1	-
	Yes	5142 (10.59)	108 (10.12)	0.96 [0.78–1.18]	0.68	0.60 [0.15–2.43]	0.47
Breach							
	No	44280 (91.15)	971 (91.00)	1	-	1	-
	Yes	3357 (6.91)	70 (6.56)	0.95 [0.73–1.25]	0.72	3.69 [0.36–37.29]	0.27

^†^ Adjusted odds ratios: adjusted for age, gender, ethnicity and employment status.

### Health needs

The health needs of veterans and non-veterans are shown in Table 3. Veterans were more likely than non-veterans to be recorded as having any mental health problem (OR = 1.47, 95% CI 1.18–1.82), harmful (OR = 1.28, 95% CI 1.08–1.51) or hazardous drinking (OR = 1.44, 95% CI 1.17–1.76), as well as being more likely to report physical health problems (OR = 2.03, 95% CI 1.52–2.72). Veterans were less likely than non-veterans to report substance misuse (OR = 0.49, 95% CI 0.40–0.60), learning difficulties (OR = 0.30, 95% CI 0.17–0.55), or social and communication difficulties (OR = 0.64, 95% CI 0.45–0.92). All of these health needs remained independently associated with veteran status in the multivariate models (see Table 3), except for alcohol misuse. Only hazardous drinking remained independently associated with veteran status (aOR = 1.37, 95% CI 1.09–1.71).

**Table 3:** Association of health needs with veteran status.

		Non-veterans (N = 48,578)	Veterans (N = 1,067)				
		N (%)	N (%)	OR [95% CI]	p	aOR [95% CI]^†^	p
Mental Health need							
	No	11711 (24.11)	203 (19.03)	1	-	1	-
	Yes	28820 (59.33)	733 (68.70)	1.47 [1.18–1.82]	<0.01	1.70 [1.36–2.12]	<0.01
Physical need							
	No	34248 (70.50)	689 (64.57)	1	-	1	-
	Yes	4646 (9.56)	190 (17.81)	2.03 [1.52–2.72]	<0.01	1.65 [1.27–2.13]	<0.01
Learning Difficulties							
	No	37971 (78.17)	922 (86.41)	1	-	1	-
	Yes	1903 (3.92)	14 (1.31)	0.30 [0.17–0.55]	<0.01	0.37 [0.19–0.72]	<0.01
Social & communication difficulties							
	No	37905 (78.03)	897 (84.07)	1	-	1	-
	Yes	1846 (3.80)	28 (2.62)	0.64 [0.45–0.92]	0.02	0.64 [0.43–0.95]	0.03
Alcohol Use							
	No	24165 (49.74)	516 (48.36)	1	-	1	-
	Harmful	6180 (12.72)	169 (15.84)	1.28 [1.08–1.51]	<0.01	1.17 [0.98–1.39]	0.08
	Hazardous	3818 (7.86)	117 (10.97)	1.44 [1.17–1.76]	<0.01	1.39 [1.10–1.76]	0.01
	Dependence	4317 (8.89)	116 (10.87)	1.26 [0.96–1.65]	0.09	1.08 [0.86–1.34]	0.51
Substance Use							
	No	24603 (50.65)	709 (66.45)	1	-	1	-
	Yes	13635 (28.07)	193 (18.09)	0.49 [0.40–0.60]	<0.01	0.62 [0.50–0.76]	<0.01

† Adjusted odds ratios: adjusted for age, gender, ethnicity and employment status.

#### Mental health

Veterans were more likely than non-veterans to be recorded as having an anxiety disorder (OR = 4.08, 95% CI 3.32–5.01), depression (OR = 1.22, 95% CI 1.04–1.44), and dementia (OR = 6.75, 95% CI 4.06–11.22). In addition, veterans were more likely to have multiple mental health problems (OR = 1.38, 95% CI 1.27–1.50). Veterans were less likely than non-veterans to be recorded as having personality disorder (OR = 0.59, 95% CI 0.46–0.78), schizophrenia (OR = 0.35, 95% CI 0.23–0.52), and ADHD (OR = 0.15, 95% CI 0.07–0.36). All of these mental health problems remained independently associated with veteran status, after adjusting for age, gender, ethnicity and employment status, except for personality disorder and depression (see Table 4).

**Table 4:** Association of mental health needs with veteran status.

		Non-veterans (N = 48,578)	Veterans (N = 1,067)				
		N (%)	N (%)	OR [95% CI]	p	aOR [95% CI]^†^	p
Any disorder							
	No	11711 (24.11)	203 (19.03)	1	-	1	-
	Yes	28820 (59.33)	733 (68.70)	1.47 [1.18–1.82]	<0.01	1.70 [1.36–2.13]	0.01
Schizophrenia							
	No	34604 (71.23)	883 (82.76)	1	-	1	-
	Yes	5927 (12.20)	53 (4.97)	0.35 [0.23–0.52]	<0.01	0.37 [0.25–0.53]	0.01
Anxiety							
	No	34491 (71.00)	546 (51.17)	1	-	1	-
	Yes	6040 (12.43)	390 (36.55)	4.08 [3.32–5.01]	<0.01	4.34 [3.45–5.45]	0.01
Personality Disorder							
	No	35093 (72.24)	857 (80.32)	1	-	1	-
	Yes	5438 (11.19)	79 (7.40)	0.59 [0.46–0.78]	<0.01	0.80 [0.61–1.04]	0.10
Bipolar							
	No	38889 (80.05)	903 (84.63)	1	-	1	-
	Yes	1642 (3.38)	33 (3.09)	0.87 [0.58–1.29]	0.48	0.79 [0.50–1.27]	0.33
Depression							
	No	27403 (56.41)	590 (55.30)	1	-	1	-
	Yes	13128 (27.02)	346 (32.43)	1.22 [1.04–1.44]	0.02	1.18 [0.99–1.41]	0.06
Dementia							
	No	40440 (83.25)	922 (86.41)	1	-	1	-
	Yes	91 (0.19)	14 (1.31)	6.75 [4.06–11.22]	<0.01	1.77 [1.01–3.12]	0.05
ADHD							
	No	39164 (80.62)	931 (87.25)	1	-	1	-
	Yes	1367 (2.81)	5 (0.47)	0.15 [0.07–0.36]	<0.01	0.25 [0.10–0.61]	0.01
Adjustment Disorder							
	No	37876 (77.97)	859 (80.51)	1	-	1	-
	Yes	2655 (5.47)	77 (7.22)	1.28 [0.98–1.68]	0.07	1.21 [0.93–1.57]	0.15
Brain Injury							
	No	40273 (82.90)	925 (86.69)	1	-	1	-
	Yes	258 (0.53)	11 (1.03)	1.86 [0.96–3.59]	0.07	1.43 [0.66–3.08]	0.36
Organic Disorder							
	No	40385 (83.13)	929 (87.07)	1	-	1	-
	Yes	146 (0.30)	7 (0.66)	2.08 [0.95–4.55]	0.07	1.20 [0.52–2.79]	0.67
Eating Disorder							
	No	40402 (83.17)	932 (87.35)	1	-	1	-
	Yes	129 (0.27)	4 (0.37)	1.34 [0.41–4.41]	0.63	1.10 [0.13–9.09]	0.93
Number of MH needs							
	1 or fewer	33525 (69.01)	690 (64.67)	1	-	1	-
	More than 1	7049 (14.51)	246 (23.06)	1.38 [1.27–1.50]	<0.01	1.48 [1.31–1.67]	0.01

† Adjusted odds ratios: adjusted for age, gender, ethnicity and employment status.

### Factors associated with different types of offending among veterans

Results of the multivariate logistic regression analyses for factors associated with each offence type among veterans are presented in the supporting information (see S1–S6 Tables).

#### Violence against the person

Age and employment status were both independently associated with violence against the person (compared to other non-violent and non-sexual offences; see S1 Table). Veterans aged 26–35 (vs. 25 and under: aOR = 0.61, 95% CI 0.37–1.00) and aged 56–65 (vs. 25 and under: aOR = 0.35, 95% CI 0.16–0.76) were less likely to have committed violence against the person than other non-violent and non-sexual offences. Veterans who were retired were more likely to have committed violence against the person than other non-violent and non-sexual offences (aOR = 2.30, 95% CI 1.05–5.04). With regard to mental health risk factors, veterans who were recorded as having an anxiety disorder (aOR = 1.42, 95% CI 1.05–1.92) were more likely to have committed violence against the person than other non-violent and non-sexual offences. Veterans who were recorded as having a problem with substance misuse (aOR = 0.51, 95% CI 0.35–0.75) were less likely to have committed violence against the person offences than other non-violent and non-sexual offences (see S2 Table). Bipolar disorder (aOR = 0.39, 95% CI 0.16–0.94) was also associated with reduced risk of violence against the person, however the low number of cases and wide confidence intervals suggest a less reliable finding (see S2 Table).

#### Acquisitive offences

Employment status and accommodation status were independently associated with acquisitive offending (vs. all other offence types; see S3 Table). Veterans who were homeless (vs. owning/renting; aOR = 2.28, 95% CI 1.27–4.10) were more likely, and veterans who were employed (vs. unemployed; aOR = 0.50, 95% CI 0.29–0.88) were less likely to have committed acquisitive offences than any other offence type. Veterans who reported bipolar disorder (aOR = 3.99, 95% CI 1.73–9.21) or substance use (aOR = 3.49, 95% CI 2.17–5.63) were more likely to have committed acquisitive offences than any other offence type. Veterans reporting alcohol misuse were less likely to have committed acquisitive offences than other offence type (aOR = 0.59, 95% CI 0.37–0.94; see S4 Table).

#### Motoring offences

Age and employment status were independently associated with motoring offences within the veteran sample (see S5 Table). Specifically, being employed (vs. unemployed; aOR = 3.70, 95% CI 2.12–6.58) was associated with motoring offences. Veterans aged 56–65 were more likely to have committed motoring offences than any other offence type (vs. 25 and under: aOR = 4.74, 95% CI 1.09–20.69), however the wide confidence intervals suggest a less reliable finding. Veterans reporting alcohol misuse were more likely to have committed motoring offences than any other offence type (aOR = 2.67, 95% CI 1.56–4.57; see S6 Table). Conversely, veterans reporting substance misuse were less likely to have committed motoring offences than veterans who had committed any other offence type (aOR = 0.32, 95% CI 0.12–0.83; see S6 Table).

## Discussion

We know that internationally a small, but significant, proportion of Armed Forces veterans become involved in the CJS after leaving service [27–29]. Research to date from the US and the UK has identified key welfare and mental health risk factors for offending among military personnel [11,30,31], but research into the needs of veterans in the CJS is lacking. Extant studies of veterans in the CJS have been limited by selected samples and limited data on health and welfare needs. This is the first UK study to utilise a national administrative database of offenders referred to a criminal justice liaison and diversion service to compare the socio-demographic characteristics, welfare needs, patterns of offending, and health and mental health needs of veterans with those of non-veteran service-users. In addition to differences in socio-demographics, key differences emerged in patterns of offending and in health and mental health needs among veterans compared to non-veterans.

There were differences in the socio-demographic characteristics of veterans compared to general population offenders: veterans were more likely to older, in employment or retired, but with just as unstable accommodation. The mean older age of veterans may be explained by military service, which delays the period during which an individual is at risk of offending in the community [11]. Higher rates of employment among veterans may result from trade or skills training acquired during military service, which may not be as available to those who have not served. The similar levels of homelessness among veterans and non-veterans suggests that military service does little to aid stability of living arrangements post-discharge [1].

An association was found between veteran status and interpersonal violence. This is consistent with previous UK and US research [9,31–33]. There are a number of potential explanations for this association. Violence by military personnel has been shown to be associated with pre-enlistment antisocial behaviour [34] and the military recruits from areas of higher social deprivation and higher crime [35]. Thus, military service may merely act to temporarily contain the behaviour of individuals already predisposed [34]. We also know that deployment, in particular combat exposure, is associated with increased risk of future violence among veterans even after adjusting for pre-military offending behaviour [9,11,31]. Furthermore, mental disorders such as PTSD, as well as alcohol misuse, have been shown to be risk factors for violence, and more general offending behaviour, among military personnel [9,11,31,33]. It is likely that a combination of these factors contributes to the overall increase in violence among veterans.

An association between veteran status and motoring offences was also found. This is consistent with evidence that road-traffic accidents are prevalent among both UK and US military personnel, and that deployment increases the likelihood of risky driving [36–38]. This may reflect a general trend towards increased risk-taking behaviour observed among military personal following return from deployment and after leaving service [39].

We observed a crude association between veteran status and sex offending. However, this association was no longer statistically significant after adjusting for socio-demographic differences. Previous studies found age-adjusted significant associations between veteran status and sex offending in UK prisons and probation services [3,6]. Our study suggests that further adjustment for differences in additional socio-demographic variables such as gender, ethnicity, and employment status accounts for this association.

Associations were shown between veteran status and anxiety disorders, dementia and multiple mental health problems. There is a wealth of evidence that links military service (in particular, combat exposure) with PTSD [40–42]. It is likely that a proportion of veterans classified as suffering with an anxiety disorder may have been suffering from PTSD symptoms, but we were unable to examine this further due to the crude categorisation of mental disorders in the dataset. Higher prevalence rates of common mental disorders, such as depression and anxiety, among Armed Forces personnel may be due to (or exacerbated by) the increased likelihood of exposure to stressors within their service roles [43], and/or the psychosocial stress associated with transition out of the military and back into civilian life [44,45]. Little is known about the prevalence of dementia in Armed Forces veterans, but a recent review suggests that veterans are at greater risk of dementia than non-veterans, perhaps due to their increased risk of other mental illnesses such as PTSD and depression [46]. We also found that non-veterans were more likely than veterans to report schizophrenia, ADHD, substance use, learning- and social/communication-difficulties. Military selection processes are likely to exclude individuals with serious mental illnesses, and the presence of these conditions would prevent someone from enlisting. This, along with the implementation of routine drug testing in the Armed Forces may account for these differences.

Veteran status was associated with hazardous (but not dependent) drinking and physical health problems. Research shows that rates of hazardous and dependent drinking among UK Armed Forces personnel are higher than among those the general population, irrespective of gender [47]. Some physical health problems among veterans are likely to be related to military service, but we were unable to establish this from the data.

Within the veteran sample, factors associated with violence against the person, acquisitive, and motoring offending were also examined. Violence against the person offences were associated with a recording of anxiety disorders (compared to other non-violent and non-sexual offences). There is a well-established link between anxiety and violence/aggression [48,49], which may be explained by deficits in emotion regulation [50,51]. Given that the veterans in the sample had a high prevalence of anxiety disorders, it is possible that the offending behaviour stems from higher levels of emotion dysregulation in veterans with anxiety. Also, PTSD was classed as an ‘anxiety disorder’ in this study’s database, which may have additionally contributed to the finding of the link between anxiety disorder and violent offending. Irritability, aggression and reckless behaviour are core symptoms of PTSD in the DSM 5 [52], and PTSD has been shown to be strongly associated with violent offending among military personnel [11,33]. Perhaps surprisingly, we did not find that alcohol misuse was associated with interpersonal violence among veterans. However, we were not comparing violent offending with non-offending in this sample. This finding tells us that veterans who misuse alcohol were no more likely to commit an offence of violence against the person than an acquisitive, or other non-violent, offence.

Acquisitive and motoring offences were associated with substance misuse and alcohol misuse, respectively (compared to all other types of offences). There is a well-established link between substance misuse and crime in general, and this is consistent across different types of substances and different types of offending [53]. Although the specific link between substance misuse and acquisitive offending has not been studied in veteran populations, our finding is unsurprising. The presence of alcohol misuse was a risk factor for motoring offences in veterans, after controlling for socio-demographic variables. This finding, again, is unsurprising given the link between alcohol consumption and risky driving behaviours [54,55].

### Strengths and limitations

A major strength of our study was the large sample size, especially of the non-veteran group, which was likely to be representative of L&D service-users. This allowed us to account for the effects of potentially confounding socio-demographic factors, which would not have been possible using a smaller sample. We were also able to directly compare veterans and non-veterans that were members of the same population–i.e. vulnerable individuals in the CJS. This is a major advantage, as any differences between them are unlikely to be biased due to different sampling methods. Furthermore, given that individuals are referred to L&D services from a range of settings, our data include individuals who had committed (or were suspected of having committed) a range of offences: from summary offences to murder and manslaughter. This increases the generalisability of our findings.

Despite these strengths, the results of our study should be interpreted in the light of a number of limitations. First, there was a significant amount of missing data. For example, 20% of cases had no veteran status, suggesting that the question is not always asked by L&D service practitioners. However, this level of missingness is not unusual among administrative datasets [56]. Furthermore, the sample size was large enough to minimise the effect of these missing data on statistical power, and enabled us to adjust for socio-demographic variables. Second, it is likely that there were arbitrary associations in the data within each L&D site due to: a) the variation in screening tools between, but not within, each site, which may have resulted in common method bias [57]; and b) common recording practices within (but not between) each site. We attempted to adjust for this clustering effect in our main analyses using methods commonly used in similar studies [26]. In addition, we were still able to demonstrate significant differences (both statistically and clinically) between veterans and non-veterans who were members of the same population (i.e. vulnerable individuals in the CJS). Third, we were unable to reliably identify repeat service-users within the database. However, our sensitivity analysis suggests that the differences we found between veterans and non-veterans are unlikely to have resulted from this issue. Fourth, the definition used by L&D services to classify whether the individual is a member of the Armed Forces is someone who has served at least one day in the UK Armed Forces. Such a definition includes individuals who may not have completed their basic training. It is likely that the number of such individuals in this dataset would be small, but we were unable to identify them. However, these individuals are currently eligible for NHS veteran community mental health services and so their inclusion in the present study is valid. Fifth, the offence types that we examined within our dataset reflect the offence-type that the individual was charged with, rather than convicted of. Similar to criminal justice systems in other parts of the world (such as the US), in the UK a charge and a conviction may not necessarily reflect the same offence (due to factors such as plea-bargaining or judicial prerogative). However, existing research suggests that arrest records (rather than conviction records) may be more reflective of the actual crime committed by the individual, as they are less likely to be affected by bureaucratic processes within the CJS [58].

### Implications

This study has highlighted the utility of using secondary routinely collected data from services engaged with offenders early in their CJS journey in order to identify those with specific needs and allow early intervention. We have identified that, among L&D service users, those who have served in the Armed Forces have offending, welfare, physical health, mental health and substance misuse needs that differ from general population offenders. In some jurisdictions in the United States of America such is the recognition of these differences in need that special veteran courts operate to divert some veterans into justice reform projects for veterans [59–61]. The creation of such courts is unlikely to be viable in countries, such as the United Kingdom, which have smaller Armed Forces. More importantly, any suggestion that former service personnel should be tried in different courts from their civilian counterparts raises the spectre of the creation of other special courts for other public servants in stressful roles, such as the police.

In other jurisdictions and countries, such as the UK, where veteran courts are not viable, the differences observed between veterans and non-veterans highlight the need for workforce training across the CJS to improve the identification of veterans and understanding of their needs within the CJS. The different pattern of mental health needs among veterans, such as the higher rate of anxiety disorders, and the links between mental disorders and offending behaviour, especially the link between anxiety disorder and interpersonal violence, emphasises the need for improved availability of psychological therapies in general in prison and the need for staff with an understanding of the impact of military service and life after the military on mental health and risk of offending. This may be especially important, given research showing that veterans tend to improve less from standard PTSD treatment approaches than non-veterans [62]. Thus, in some areas, specialist veteran in-reach services in the CJS may be a viable solution. However, where such services are not an option, an integrated approach to the delivery of mental health, substance-misuse and welfare services should be the goal, with veterans’ champions co-ordinating care to ensure the holistic needs of veterans are met and to ultimately reduce the risk of re-offending. Our results also highlight the importance of improving the assessment and treatment of trauma-related mental health problems, which would not only benefit veterans, but also the general offender population.

## Supporting information

S1 Table Association of socio-demographic variables with violence against the person offences within the veteran sample. (XLSX)

S2 Table Association of mental health needs with violence against the person offences within the veteran sample. (XLSX)

S3 Table Association of socio-demographics with acquisitive offences in the veteran sample. (XLSX)

S4 Table Association of mental health needs with acquisitive offences in the veteran sample. (XLSX)

S5 Table Association of socio-demographic variables with motoring offences within the veteran sample. (XLSX)

S6 Table Association of mental health needs with motoring offences within the veteran sample. (XLSX)
